# Xerostomia symptom severity and frailty among community-dwelling older adults: a survey-weighted structural equation modeling analysis

**DOI:** 10.3389/fdmed.2026.1941262

**Published:** 2026-10-14

**Authors:** Patrícia Soares da Silva Pereira, Billy de Jesus Vieira de Paula e Silva, Ana Laura Melo Gonçalves, Rafaela Bilancieri, Beatriz Della Terra Mouco Garrido, Roger Palma, Roosevelt Silva Bastos

**Affiliations:** 1 Bauru School of Medicine, University of São Paulo, Bauru, Brazil; 2 Department of Pediatric Dentistry, Orthodontics and Public Health, Bauru School of Dentistry, University of São Paulo, Bauru, Brazil

**Keywords:** aged, frailty, mediation analysis, nutrition status, structural equation modeling, xerostomia

## Abstract

**Objective:**

To investigate the direct and indirect associations between xerostomia symptom severity and frailty among community-dwelling older adults, considering nutritional vulnerability as a potential mediator using structural equation modeling.

**Methods:**

This cross-sectional population-based study analyzed 398 community-dwelling older adults from two municipalities in southeastern Brazil. Xerostomia was assessed using the Summated Xerostomia Inventory (SXI-5), nutritional status using the Mini Nutritional Assessment–Short Form (MNA-SF), and frailty using the Edmonton Frail Scale (EFS). Xerostomia, nutritional vulnerability, and frailty were modeled as continuous scores. Nutritional vulnerability was calculated as 14 minus the MNA-SF score, with higher values indicating poorer nutritional status. Survey-weighted structural equation modeling estimated direct, indirect, and total associations, adjusting for age, sex, and educational attainment and accounting for clustering in the sampling design.

**Results:**

The weighted prevalence of high xerostomia symptoms was 24.5% (95% CI: 16.2–35.3), nutritional risk or malnutrition was 35.5% (95% CI: 29.7–41.8), and frailty was 12.3% (95% CI: 6.5–22.1). Xerostomia symptom severity was not significantly associated with nutritional vulnerability (*B* = 0.146; 95% CI: −0.073 to 0.364; standardized *β* = 0.179; *P* = 0.177). Nutritional vulnerability was associated with higher EFS scores (*B* = 0.226; 95% CI: 0.046–0.405; *β* = 0.206; *P* = 0.017). Xerostomia symptom severity showed a significant direct association with the EFS score (*B* = 0.130; 95% CI: 0.008–0.252; *β* = 0.145; *P* = 0.039) and a significant total association (*B* = 0.163; 95% CI: 0.012–0.313; *β* = 0.182; *P* = 0.036). However, the indirect association through nutritional vulnerability was not statistically significant (*B* = 0.033; 95% CI: −0.022 to 0.088; *β* = 0.037; *P* = 0.225). The model explained 9.2% of the variance in nutritional vulnerability and 15.3% of the variance in frailty.

**Conclusions:**

Greater xerostomia symptom severity was directly associated with higher frailty scores, but there was no evidence of an indirect association through nutritional vulnerability. These findings indicate that the relationship between xerostomia and frailty may involve pathways beyond nutritional status and reinforce the potential value of integrating oral health assessment into comprehensive care for older adults.

## Introduction

Frailty is an age-related clinical syndrome characterized by diminished physiological reserve and increased vulnerability to adverse health outcomes following relatively minor stressors (1, 2). As one of the most important consequences of population ageing, frailty has become an increasing public health concern worldwide due to the rapid growth in the number of older adults living with chronic conditions and functional decline (3). Older adults with frailty are at substantially higher risk of falls, disability, hospitalization, institutionalization, and premature mortality, resulting in considerable individual, social, and healthcare burdens (1, 4). Because frailty is a dynamic and potentially preventable condition, identifying modifiable risk factors and early clinical markers has become a priority for promoting healthy ageing and reducing the burden of disability in older populations (2).

Growing evidence suggests that oral health is an important component of healthy ageing and may influence the development and progression of frailty. Several epidemiological studies have reported that poor oral health, including tooth loss, impaired oral function, and xerostomia, is associated with an increased risk of frailty and other adverse geriatric outcomes among community-dwelling older adults (5–7). More recently, longitudinal evidence from the English Longitudinal Study of Ageing demonstrated that poor oral health predicts incident frailty over a 13-year follow-up, reinforcing the importance of oral conditions as determinants of healthy ageing (8). Among these conditions, xerostomia has consistently emerged as one of the oral symptoms most strongly associated with frailty, yet the mechanisms underlying this relationship remain poorly understood.

Xerostomia is a common oral condition among older adults, affecting approximately one in every four individuals living in the community, and is frequently associated with polypharmacy, chronic diseases, and age-related changes in salivary gland function (9–11). Saliva plays a fundamental role in maintaining oral homeostasis by facilitating mastication, bolus formation, swallowing, taste perception, and oral lubrication. Consequently, reduced salivary function may impair food intake, decrease dietary quality and protein consumption, and increase the risk of malnutrition (12). These biological mechanisms suggest that nutritional impairment may represent an important pathway linking xerostomia to frailty, although this hypothesis has received limited investigation in population-based studies.

Despite accumulating evidence linking xerostomia to frailty, important gaps remain in understanding the mechanisms underlying this association. Most previous studies have been cross-sectional and have focused primarily on estimating direct associations between xerostomia and frailty, without exploring the potential pathways through which xerostomia may be associated with greater frailty (6, 7, 13). Although nutritional impairment has been proposed as a biologically plausible mechanism linking xerostomia to frailty through impaired mastication, swallowing, and reduced dietary intake (9, 12), empirical evidence quantifying its contribution to this association remains limited. Elucidating these potential pathways may improve our understanding of the relationship between oral health and frailty while identifying opportunities for earlier intervention and prevention among older adults. Therefore, this study aimed to investigate the direct and indirect pathways linking xerostomia symptom severity to frailty among community-dwelling older adults by evaluating nutritional vulnerability as a potential mediator using survey-weighted structural equation modeling. We hypothesized that nutritional vulnerability would partially mediate the association between xerostomia and frailty.

## Methods

### Study design and setting

This cross-sectional population-based study was conducted among community-dwelling older adults residing in two municipalities in the state of São Paulo, Brazil (Bauru and Boraceia). Data were collected between March 2025 and April 2026 as part of a broader research project investigating oral health, nutrition, and healthy ageing among older adults. The study was designed to examine the relationships between oral health conditions and geriatric outcomes in community settings.

The study was approved by the Research Ethics Committee of the Bauru School of Dentistry, University of São Paulo (CAAE: 79324524.6.0000.5417; approval no. 7.034.887), and all participants provided written informed consent prior to data collection. The study was conducted in accordance with the ethical principles of the Declaration of Helsinki.

### Participants and sampling

A multistage, population-based sampling design was adopted to obtain a representative sample of older adults residing in the study area. Primary sampling units consisted of public primary healthcare catchment areas within the Brazilian Unified Health System, including Family Health Strategy units (USF) and Primary Health Care Centers (UBS), while older adults living within these catchment areas were selected as secondary sampling units through simple random sampling. The sampling design included 17 primary sampling units across the two municipalities. Eligible participants were aged 60 years or older, had been living in the selected catchment area for at least one year, and were able to provide informed consent. The initial study sample comprised 437 community-dwelling older adults, of whom 398 (91.1%) had complete information for all variables included in the structural equation model and constituted the final analytical sample. Sampling weights were calculated to account for the multistage sampling design and unequal probabilities of selection. The final weights incorporated the sampling fraction within each primary sampling unit and were calibrated according to the age and sex distribution of the target population. All descriptive and structural equation modeling analyses accounted for sampling weights and clustering at the primary sampling unit level to obtain population-representative estimates for the study area.

### Xerostomia

Xerostomia was assessed using the Summated Xerostomia Inventory (SXI-5) (9), a validated self-reported instrument comprising five items that assess the frequency of dry mouth symptoms. Each item is scored on a three-point Likert scale (1 = never, 2 = occasionally, and 3 = often), yielding a total score ranging from 5 to 15, with higher scores indicating greater xerostomia severity. The SXI-5 measures the subjective experience of dry mouth and does not constitute an objective assessment of salivary flow. For the primary structural equation model, the total SXI-5 score was analyzed as a continuous variable. For descriptive analyses only, participants were categorized as having low xerostomia symptoms (SXI-5 <9) or high xerostomia symptoms (SXI-5 ≥9) using an *a priori* operational threshold. Because no universally established diagnostic cutoff is available for the SXI-5, these categories were used to describe symptom burden and were not interpreted as clinical diagnoses.

### Frailty

Frailty was assessed using the Edmonton Frail Scale (EFS) (14), a multidimensional instrument developed to evaluate frailty across multiple domains of health in older adults. The EFS comprises 11 items covering nine domains, including cognition, general health status, functional independence, social support, medication use, nutrition, mood, continence, and functional performance. Total scores range from 0 to 17, with higher scores indicating greater frailty. The instrument has demonstrated good validity and reliability for assessing frailty in community-dwelling older adults and has been culturally adapted and validated for use in Brazil (15). Although the EFS allows classification into categories ranging from no frailty to severe frailty, the total score was analyzed as a continuous variable in the primary structural equation model to preserve statistical information and maximize analytical power. For descriptive purposes, frailty was also categorized using the conventional cutoff of EFS ≥7 to indicate the presence of frailty. Because the EFS contains a nutritional component that could conceptually overlap with the MNA-SF, a sensitivity analysis was performed using a modified EFS score from which the nutritional component was excluded. This modified score was analyzed as a continuous outcome.

### Nutritional status

Nutritional status was assessed using the Mini Nutritional Assessment–Short Form (MNA-SF) (16), a validated screening instrument specifically developed for older adults. The MNA-SF comprises six items evaluating food intake, recent weight loss, mobility, psychological stress or acute disease, neuropsychological problems, and body mass index or calf circumference. In the present study, calf circumference was used for the sixth item and scored according to the standard MNA-SF criteria: 0 points for values <31 cm and 3 points for values ≥31 cm. Total scores range from 0 to 14, with higher scores indicating better nutritional status. According to the standard classification, participants were categorized as well nourished (12–14 points), at risk of malnutrition (8–11 points), or malnourished (0–7 points). For descriptive comparisons, participants at risk of malnutrition and those classified as malnourished were combined into a nutritional risk/malnutrition category (MNA-SF <12). For the primary structural equation model, nutritional vulnerability was operationalized as 14 minus the total MNA-SF score and analyzed continuously, so that higher values indicated poorer nutritional status. Nutritional vulnerability was specified as the potential mediator in the structural equation model.

### Covariates

Age, sex, and educational attainment were included as covariates based on their established associations with xerostomia, nutritional status, and frailty. Age was analyzed as a continuous variable, sex was categorized as male or female, and educational attainment was classified as up to elementary education, secondary education, and higher education or above. In the structural equation model, sex was represented by a binary indicator, with female as the reference category. Educational attainment was represented by two indicator variables for secondary education and higher education or above, with up to elementary education as the reference category. Age, sex, and educational attainment were included as predictors of both nutritional vulnerability and the EFS score.

### Statistical analysis

Descriptive analyses were performed to characterize the study population according to sociodemographic characteristics, xerostomia symptom severity, nutritional status, and frailty. Categorical variables were summarized using survey-weighted proportions with 95% confidence intervals (95% CIs), and continuous variables using survey-weighted means with corresponding 95% CIs. Unweighted absolute frequencies were also reported. Prevalence estimates and their 95% CIs were calculated on the logit scale. All descriptive analyses accounted for sampling weights and clustering at the primary sampling unit level. The completeness of the study variables and patterns of missing information were examined descriptively.

The prevalence of frailty, defined as an Edmonton Frail Scale (EFS) score ≥7, was estimated according to xerostomia symptom category, nutritional status, age group, sex, and educational attainment. Differences in frailty prevalence were evaluated using Pearson chi-square tests with Rao–Scott correction for the survey design, reported as design-based F statistics. The weighted mean EFS score and the prevalence of impairment in each EFS component were also estimated overall and according to xerostomia symptom category. For individual EFS components, any impairment was defined as a component score ≥1. Differences in the mean EFS score according to xerostomia symptom category were evaluated using survey-adjusted linear regression, while differences in categorical EFS components were assessed using Pearson chi-square tests with Rao–Scott correction, reported as design-based F statistics.

Structural equation modeling (SEM) was used to examine the hypothesized pathways linking xerostomia symptom severity, nutritional vulnerability, and frailty severity. Based on an *a priori* conceptual model, nutritional vulnerability was specified as a potential mediator of the association between xerostomia and frailty, while age, sex, and educational attainment were included as exogenous covariates predicting both the mediator and the outcome (Figure 1). The SXI-5 score, nutritional vulnerability score, and EFS score were analyzed as continuous variables. The model simultaneously estimated the direct association between xerostomia and frailty and the indirect association through nutritional vulnerability**.** The SEM was estimated in the complete-case analytical sample using sampling weights and linearized standard errors, accounting for clustering at the primary sampling unit level. Unstandardized coefficients (B) with 95% CIs and standardized path coefficients (β) were reported. Direct, indirect, and total effects were estimated, with indirect effects calculated as the products of the corresponding path coefficients, and the proportion of variance explained in nutritional vulnerability and frailty was summarized using R^2^. Because the specified path model was just-identified, global model-fit indices were not interpreted. Two sensitivity analyses were conducted: the first additionally adjusted both model equations for municipality, and the second used a modified EFS score excluding its nutritional component. Statistical significance was defined as a two-sided *P* < 0.05. All analyses were conducted using Stata version 18 (StataCorp LLC, College Station, TX, USA); SEM was performed using the sem command with the svy prefix.

**Figure 1:**
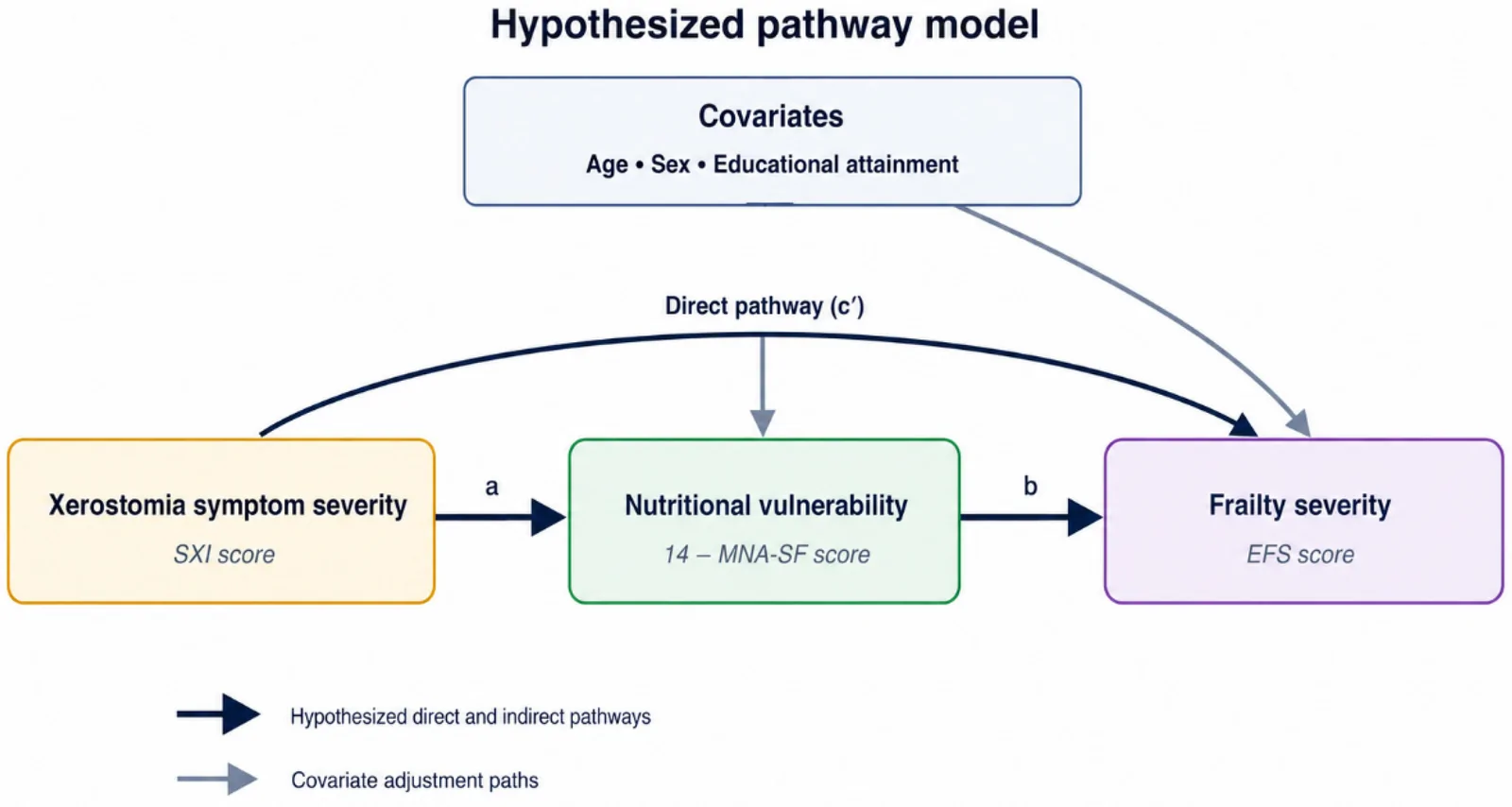
Hypothesized pathway model linking xerostomia symptom severity, nutritional vulnerability, and frailty severity. The model included a direct pathway from xerostomia symptom severity, measured using the Summated Xerostomia Inventory (SXI-5), to frailty severity, measured using the Edmonton Frail Scale (EFS), and a hypothesized indirect pathway through nutritional vulnerability. Nutritional vulnerability was operationalized as 14 minus the Mini Nutritional Assessment–Short Form (MNA-SF) score, so that higher values indicated poorer nutritional status. Age, sex, and educational attainment were included as covariates of the mediator and outcome. Path *a* represents the association between xerostomia symptom severity and nutritional vulnerability; path *b* represents the association between nutritional vulnerability and frailty severity; and path *c'* represents the direct association between xerostomia symptom severity and frailty severity after accounting for nutritional vulnerability. The indirect effect was estimated as the product of paths *a* and *b*.

## Results

The initial study sample comprised 437 community-dwelling older adults, of whom 398 (91.1%) had complete information for all variables included in the structural equation model and constituted the final analytical sample. Thirty-nine participants (8.9%) were excluded because of incomplete information. The weighted mean age was 71.13 years (95% CI: 70.28–71.99), and 59.9% (95% CI: 58.0–61.8) were women. Up to elementary education was reported by 68.3% (95% CI: 52.7–80.7), secondary education by 21.2% (95% CI: 13.6–31.5), and higher education or above by 10.4% (95% CI: 5.1–20.1). High xerostomia symptoms were present in 24.5% (95% CI: 16.2–35.3) of participants, and 35.5% (95% CI: 29.7–41.8) were classified as being at nutritional risk or malnourished. The weighted mean Edmonton Frail Scale score was 3.66 (95% CI: 3.26–4.07), and 12.3% (95% CI: 6.5–22.1) of participants were classified as frail. The prevalence of frailty was higher among participants with high xerostomia symptoms than among those with low xerostomia symptoms: 26.5% (95% CI: 12.6–47.3) versus 7.7% (95% CI: 3.7–15.6), respectively (*P* = 0.001). Frailty prevalence did not differ significantly between participants at nutritional risk or with malnutrition and those with normal nutritional status: 15.8% (95% CI: 8.1–28.7) versus 10.4% (95% CI: 4.1–23.7), respectively (*P* = 0.351). Frailty prevalence differed according to educational attainment (*P* = 0.002) and age group (*P* = 0.004), whereas no statistically significant difference was observed by sex (*P* = 0.277) (Table 1). The education-specific estimates did not indicate a monotonic gradient and should be interpreted cautiously because of the smaller subgroup sizes and limited precision of some estimates.

**Table 1:** Prevalence of frailty according to xerostomia, nutritional status, and sociodemographic characteristics among community-dwelling older adults (*n* = 398).

Characteristics	n	Frailty % (95% CI)	*p*-value
Xerostomia symptoms			
High	98	26.5 (12.6–47.3)	**0**.**001**
Low	300	7.7 (3.7–15.6)	
Nutritional status			
Normal	260	10.4 (4.1–23.7)	0.351
At risk of malnutrition/malnourished	138	15.8 (8.1–28.7)	
Sex			
Female	241	14.4 (7.2–26.8)	0.277
Male	157	9.1 (3.6–21.1)	
Educational attainment			
Up to elementary education	273	16.6 (8.3–30.3)	**0**.**002**
Secondary education	74	1.0 (0.1–8.7)	
Higher education or above	51	7.3 (2.8–17.3)	
Age group			
60–69 years	165	9.2 (3.5–21.9)	**0**.**004**
70–79 years	164	20.4 (11.4–34.0)	
≥80 years	69	2.9 (0.9–8.4)	

Total analytical sample: *n* = 398. Values of n are unweighted counts. Prevalence estimates and 95% confidence intervals were weighted and accounted for the complex survey design. *P*-values were obtained using the Pearson chi-square test with Rao–Scott correction for the survey design, reported as a design-based F statistic.

Bold values indicate statistically significant results (*p* < 0.05).

Participants with high xerostomia symptoms had a higher weighted mean EFS score than those with low xerostomia symptoms: 4.74 (95% CI: 4.00–5.48) versus 3.32 (95% CI: 2.89–3.74), respectively, with a mean difference of 1.42 points (95% CI: 0.68–2.17; *P* = 0.001). High xerostomia symptoms were also associated with a higher prevalence of fair or poor self-rated health [69.6% (95% CI: 56.7–80.0) vs. 45.5% (95% CI: 30.8–61.1); *P* = 0.001], multiple medication use [51.7% (95% CI: 33.6–69.3) vs. 30.5% (95% CI: 21.7–40.9); *P* = 0.036], depressed mood [50.8% (95% CI: 35.4–66.1) vs. 30.8% (95% CI: 18.9–45.9); *P* = 0.014], and incontinence [45.9% (95% CI: 29.9–62.8) vs. 27.6% (95% CI: 18.9–38.4); *P* = 0.013]. No statistically significant differences were observed for the other EFS components (all *P* ≥ 0.171) (Table 2).

**Table 2:** Edmonton frail scale score and components according to xerostomia status among community-dwelling older adults.

EFS measure	Overall, % (95% CI)	High xerostomia, % (95% CI)	Low xerostomia, % (95% CI)	*P*-value
Total EFS score, mean (95% CI)	3.66 (3.26–4.07)	4.74 (4.00–5.48)	3.32 (2.89–3.74)	**0**.**001**
Cognitive impairment	18.7 (11.1–29.6)	25.1 (10.5–48.8)	16.6 (9.5–27.2)	0.295
Hospital admission in the previous year	13.8 (9.4–19.8)	13.9 (7.2–25.1)	13.8 (7.8–23.2)	0.986
Fair or poor self-rated health	51.4 (37.8–64.9)	69.6 (56.7–80.0)	45.5 (30.8–61.1)	**0**.**001**
Functional dependence	47.3 (32.2–62.9)	55.7 (37.5–72.6)	44.5 (28.6–61.6)	0.217
Insufficient social support	10.7 (7.0–16.1)	14.6 (6.3–30.6)	9.4 (5.2–16.5)	0.398
Multiple medication use	35.7 (27.1–45.3)	51.7 (33.6–69.3)	30.5 (21.7–40.9)	**0**.**036**
Nutritional impairment	26.8 (19.4–35.8)	36.6 (20.4–56.4)	23.7 (16.0–33.5)	0.171
Depressed mood	35.7 (24.8–48.3)	50.8 (35.4–66.1)	30.8 (18.9–45.9)	**0**.**014**
Incontinence	32.1 (23.2–42.5)	45.9 (29.9–62.8)	27.6 (18.9–38.4)	**0**.**013**
Impaired functional performance	33.8 (23.3–46.1)	33.9 (17.3–55.8)	33.7 (23.6–45.7)	0.984
Frailty, EFS ≥7	12.3 (6.5–22.1)	26.5 (12.6–47.3)	7.7 (3.7–15.6)	**0**.**001**

Total analytical sample: *n* = 398. EFS, Edmonton Frail Scale; CI, confidence interval. Values are survey-weighted means or prevalence estimates with 95% confidence intervals. For individual EFS components, impairment was defined as a component score ≥1. The *P*-value for the total EFS score was obtained using survey-adjusted linear regression. *P*-values for the individual components and frailty were obtained using the Pearson chi-square test with Rao–Scott correction for the survey design, reported as a design-based F statistic.

Bold values indicate statistically significant results (*p* < 0.05).

Structural equation modeling showed that greater xerostomia symptom severity was not significantly associated with nutritional vulnerability (*B* = 0.146; 95% CI: −0.073 to 0.364; standardized *β* = 0.179; *P* = 0.177). Nutritional vulnerability was positively associated with the EFS score (*B* = 0.226; 95% CI: 0.046–0.405; *β* = 0.206; *P* = 0.017). Greater xerostomia symptom severity was directly associated with a higher EFS score (*B* = 0.130; 95% CI: 0.008–0.252; *β* = 0.145; *P* = 0.039). However, the indirect association through nutritional vulnerability was not statistically significant (*B* = 0.033; 95% CI: −0.022 to 0.088; *β* = 0.037; *P* = 0.225). The total association between xerostomia symptom severity and the EFS score was statistically significant (*B* = 0.163; 95% CI: 0.012–0.313; *β* = 0.182; *P* = 0.036). The model explained 9.2% of the variance in nutritional vulnerability and 15.3% of the variance in the EFS score (Table 3 and Figure 2).

**Table 3:** Direct, indirect, and total associations from the structural equation model linking xerostomia symptom severity, nutritional vulnerability, and frailty severity among community-dwelling older adults.

Pathway	B	95% CI	Standardized β	*P*-value
Direct effects on nutritional vulnerability				
SXI score → nutritional vulnerability	0.146	−0.073 to 0.364	0.179	0.177
Age → nutritional vulnerability	−0.016	−0.057 to 0.024	−0.060	0.408
Male vs. female → nutritional vulnerability	−0.788	−1.161 to −0.416	−0.195	**<0**.**001**
Secondary vs. up to elementary education → nutritional vulnerability	−0.145	−0.819 to 0.529	−0.030	0.654
Higher vs. up to elementary education → nutritional vulnerability	−0.225	−1.144 to 0.693	−0.035	0.610
Direct effects on frailty				
Nutritional vulnerability → EFS score	0.226	0.046 to 0.405	0.206	**0**.**017**
SXI score → EFS score	0.130	0.008 to 0.252	0.145	**0**.**039**
Age → EFS score	0.008	−0.044 to 0.060	0.028	0.742
Male vs. female → EFS score	−0.050	−0.749 to 0.648	−0.011	0.881
Secondary vs. up to elementary education → EFS score	−1.086	−1.978 to −0.195	−0.205	**0**.**020**
Higher vs. up to elementary education → EFS score	−0.928	−1.868 to 0.012	−0.131	0.053
Indirect effects through nutritional vulnerability				
SXI score → nutritional vulnerability → EFS score	0.033	−0.022 to 0.088	0.037	0.225
Age → nutritional vulnerability → EFS score	−0.004	−0.014 to 0.007	−0.012	0.475
Male vs. female → nutritional vulnerability → EFS score	−0.178	−0.354 to −0.002	−0.040	**0**.**048**
Secondary vs. up to elementary education → nutritional vulnerability → EFS score	−0.033	−0.178 to 0.113	−0.006	0.640
Higher vs. up to elementary education → nutritional vulnerability → EFS score	−0.051	−0.234 to 0.132	−0.007	0.564
Total effects on frailty				
SXI score → EFS score	0.163	0.012 to 0.313	0.182	**0**.**036**
Age → EFS score	0.005	−0.044 to 0.053	0.015	0.845
Male vs. female → EFS score	−0.228	−0.812 to 0.356	−0.052	0.420
Secondary vs. up to elementary education → EFS score	−1.119	−2.051 to −0.187	−0.211	**0**.**022**
Higher vs. up to elementary education → EFS score	−0.979	−1.908 to −0.049	−0.138	**0**.**040**

Total analytical sample: *n* = 398. B, unstandardized coefficient; β, standardized coefficient; CI, confidence interval; EFS, Edmonton frail scale; SXI, summated xerostomia inventory. Higher SXI scores indicate greater xerostomia symptom severity. Nutritional vulnerability was calculated as 14 minus the Mini Nutritional Assessment–Short Form score, so higher values indicate poorer nutritional status. Age was entered continuously. Sex was coded as female = 0 and male = 1. Educational attainment was represented by two indicator variables, with up to elementary education as the reference category. Indirect effects were calculated as the products of the corresponding pathway coefficients. The model was estimated for complete cases using survey weights and linearized standard errors, accounting for the clustering of the sampling design. The model explained 9.2% of the variance in nutritional vulnerability and 15.3% of the variance in the EFS score.

**Figure 2:**
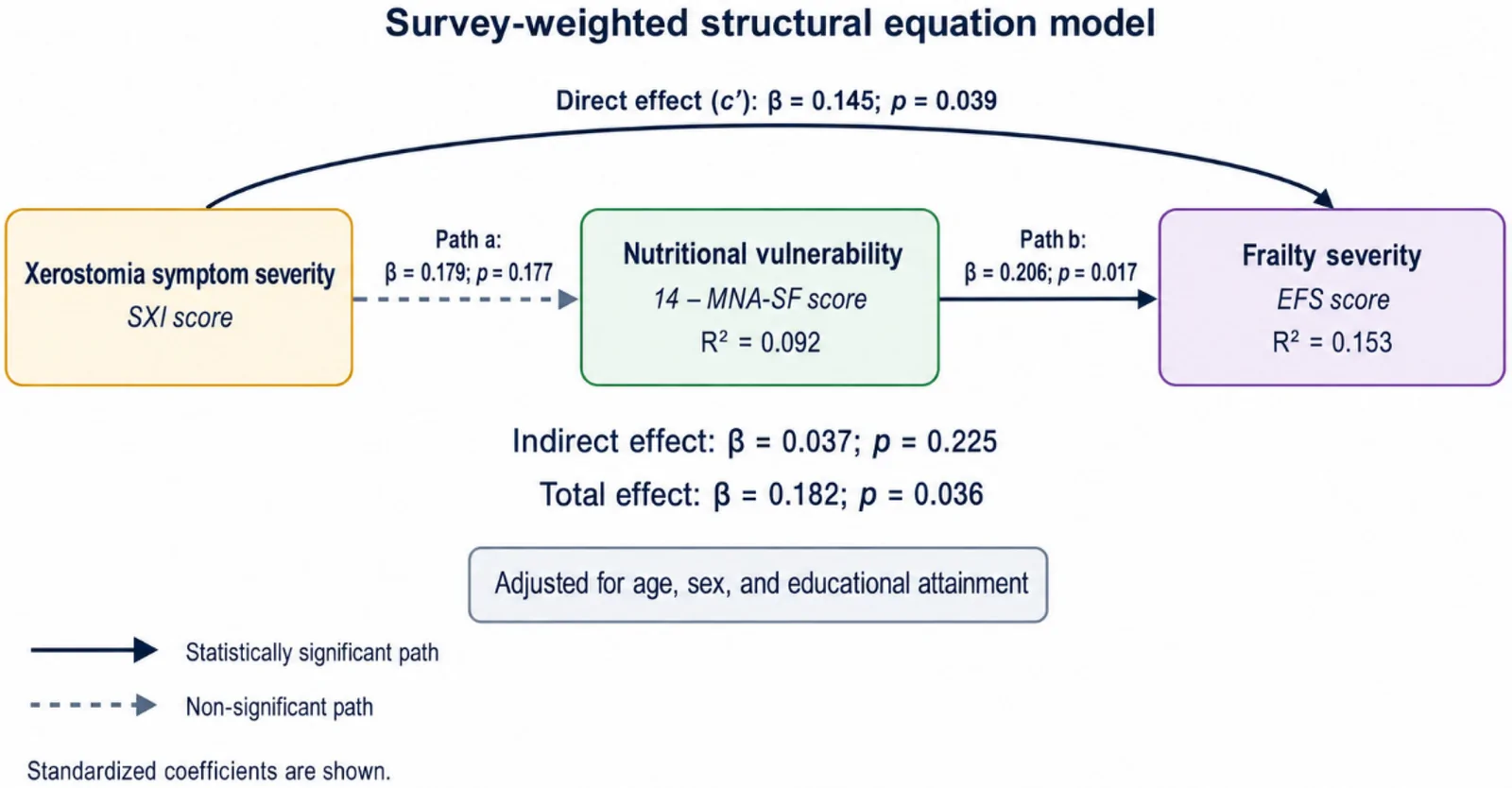
Survey-weighted structural equation model linking xerostomia symptom severity, nutritional vulnerability, and frailty severity. Standardized path coefficients are presented. The model was adjusted for age, sex, and educational attainment. The solid arrows indicate statistically significant paths, whereas the dashed arrow indicates a non-significant path retained in the hypothesized model. Higher SXI scores indicate greater xerostomia symptom severity. Nutritional vulnerability was operationalized as 14 minus the MNA-SF score, so that higher values indicate poorer nutritional status. The direct association between xerostomia symptom severity and the EFS score was statistically significant (*β* = 0.145; *P* = 0.039), whereas the indirect association through nutritional vulnerability was not significant (*β* = 0.037; *P* = 0.225). The total association was *β* = 0.182 (*P* = 0.036). Values of R^2^ indicate the proportion of variance explained for each endogenous variable. The model accounted for the sampling weights and clustering of the survey design.

Sensitivity analyses supported the robustness of the direct association. After additional adjustment for municipality, the direct association between xerostomia symptom severity and the EFS score remained statistically significant (*B* = 0.128; 95% CI: 0.002–0.254; *β* = 0.144; *P* = 0.047), as did the total association (*B* = 0.162; 95% CI: 0.009–0.316; *β* = 0.182; *P* = 0.040), whereas the indirect association remained non-significant (*B* = 0.034; 95% CI: −0.022 to 0.090; *β* = 0.038; *P* = 0.214) (Supplementary Table S1). When the nutritional component was excluded from the EFS score, the direct association (*B* = 0.123; 95% CI: 0.014–0.231; *β* = 0.142; *P* = 0.029) and total association (*B* = 0.141; 95% CI: 0.021–0.262; *β* = 0.163; *P* = 0.024) remained statistically significant. In contrast, the association between nutritional vulnerability and the modified EFS score was attenuated and no longer statistically significant (*B* = 0.129; 95% CI: −0.043 to 0.302; *β* = 0.122; *P* = 0.132), and the indirect association remained non-significant (*B* = 0.019; 95% CI: −0.021 to 0.058; *β* = 0.022; *P* = 0.328) (Supplementary Table S2). The extent and patterns of missing information and the derivation of the final analytical sample are presented in Supplementary Table S3.

## Discussion

This population-based study extends current evidence on the relationship between xerostomia and frailty by examining nutritional vulnerability as a potential indirect pathway among community-dwelling older adults. Consistent with part of our hypothesis, participants with high xerostomia symptoms had higher frailty scores and a higher prevalence of impairment in several EFS components, particularly self-rated health, medication use, mood, and continence. Structural equation modeling showed that greater xerostomia symptom severity was directly associated with higher frailty scores after accounting for age, sex, and educational attainment. However, the indirect association through nutritional vulnerability was not statistically significant. The total association remained statistically significant. These findings do not support nutritional vulnerability as a mediator in the present study, suggest that other pathways may contribute to the association between xerostomia and frailty, and reinforce the multifactorial nature of frailty in later life.

The present findings are consistent with previous studies reporting an association between xerostomia and frailty among older adults. Cross-sectional studies conducted in Japan and Mexico have consistently shown that older adults with dry mouth are more likely to present physical frailty or higher frailty scores, even after adjustment for sociodemographic and health-related factors (5–7). More recently, longitudinal evidence from the English Longitudinal Study of Ageing demonstrated that poor oral health was associated with the subsequent development of frailty, reinforcing the importance of oral conditions in healthy ageing (8). While these studies established a consistent epidemiological association between oral health and frailty, they provided limited insight into the mechanisms underlying this relationship. Our findings extend this body of evidence by evaluating nutritional vulnerability as a potential pathway in the association between xerostomia symptom severity and frailty. However, the indirect association was not statistically significant, and this potential pathway requires further investigation in longitudinal studies.

Several biological mechanisms may explain why nutritional vulnerability was considered a potential pathway in the association between xerostomia and frailty. Saliva plays a fundamental role in maintaining oral function by facilitating lubrication, mastication, bolus formation, swallowing, taste perception, and the initial stages of digestion (9). Consequently, dry-mouth symptoms may compromise food intake, decrease dietary variety and protein intake, and increase the risk of malnutrition (12). Xerostomia is a subjective symptom and may occur with or without objectively reduced salivary flow, which was not measured in the present study. Poor nutritional status, in turn, has been consistently associated with loss of muscle mass and strength, impaired physical performance, and progression to frailty among older adults (2). Consistent with this biological framework, greater nutritional vulnerability was associated with higher frailty scores. Nevertheless, xerostomia symptom severity was not significantly associated with nutritional vulnerability, and the indirect association was not statistically significant. Thus, although nutritional vulnerability remains biologically plausible, the present findings do not support it as a mediator of the association between xerostomia and frailty. Because the MNA-SF captures multiple dimensions of nutritional vulnerability, including recent food intake, weight loss, mobility, and body mass index or calf circumference, future studies should examine whether specific nutritional dimensions are associated with xerostomia symptom severity rather than assuming a cumulative indirect pathway. This possibility is also supported by previous studies suggesting that impaired oral function contributes to nutritional vulnerability and functional decline among older adults (5, 17).

The persistence of a significant direct association between xerostomia symptom severity and frailty after accounting for nutritional vulnerability suggests that additional pathways not captured in the present model are also likely to contribute to this association. Xerostomia frequently coexists with other oral conditions, including impaired chewing ability, swallowing difficulties, reduced oral function, tooth loss, and oral pain, all of which may independently influence physical function and frailty (5, 6, 13, 17). Furthermore, xerostomia is often associated with polypharmacy and multiple chronic diseases, conditions that are themselves strongly associated with frailty in older adults (10, 18–20). Consistent with these potential pathways, participants with high xerostomia symptoms had a higher prevalence of multiple medication use, poorer self-rated health, depressed mood, and incontinence. These findings reinforce the multifactorial nature of frailty and suggest that a broader network of oral, biological, functional, and clinical factors may link xerostomia to frailty. Although nutritional vulnerability is one biologically plausible component of this network, its mediating role was not supported in the present study.

The present findings also have potential clinical and public health implications. Xerostomia is a common symptom among older adults, can be assessed using brief validated questionnaires, and can be readily identified during dental and primary healthcare consultations. Although the cross-sectional design precludes causal inference, the observed direct association between xerostomia symptom severity and frailty suggests that older adults reporting xerostomia may benefit from a more comprehensive assessment of nutritional status and frailty. Although the indirect association through nutritional vulnerability was not statistically significant, nutritional assessment may remain clinically relevant because of the broader nutritional consequences potentially associated with dry-mouth symptoms. These findings reinforce the importance of integrated care involving dental professionals, physicians, nutritionists, and other members of multidisciplinary teams to identify older adults with complex oral and nutritional needs. From a public health perspective, incorporating oral symptoms such as xerostomia into comprehensive geriatric assessments may contribute to the recognition of vulnerable individuals and support strategies aimed at promoting healthy ageing.

This study has several strengths. It was based on a population-based sample of community-dwelling older adults, and all descriptive and structural equation modeling analyses accounted for the complex sampling design, enhancing the representativeness of the estimates for the study area. In addition, validated instruments were used to assess xerostomia symptom severity, nutritional status, and frailty, and structural equation modeling allowed the simultaneous evaluation of direct and indirect associations within a theoretically grounded framework. The direct association remained significant after additional adjustment for municipality and when the nutritional component was excluded from the EFS, supporting the robustness of this finding. Nevertheless, some limitations should be considered. First, the cross-sectional design precludes establishing temporal relationships or causal inferences. Second, xerostomia was assessed using a self-reported instrument rather than objective measures of salivary flow. However, xerostomia and hyposalivation are distinct constructs, and the SXI-5 was appropriate for assessing the subjective severity of dry-mouth symptoms. Third, the EFS includes a nutritional component that may partially overlap conceptually with the MNA-SF and may have increased the observed association between nutritional vulnerability and frailty. When this component was excluded from the EFS, the association between nutritional vulnerability and frailty was attenuated and was no longer statistically significant, indicating that the primary association between these constructs should be interpreted cautiously. Fourth, missing information was present for some variables, and the analyses were restricted to 398 participants with complete data, corresponding to 91.1% of the initial sample. Complete-case analysis may introduce selection bias if data are not missing completely at random. Finally, although the analyses were adjusted for age, sex, and educational attainment, residual confounding by unmeasured factors such as chronic diseases, polypharmacy, and other oral conditions cannot be excluded. Because the study was conducted in two municipalities in southeastern Brazil, the generalizability of the findings to other populations may also be limited. Future longitudinal studies are needed to examine the temporal direction of the observed associations and investigate other potential mechanisms linking xerostomia, nutritional vulnerability, and frailty.

## Conclusion

Greater xerostomia symptom severity was directly associated with higher frailty scores among community-dwelling older adults, but there was no evidence of an indirect association through nutritional vulnerability. These findings do not support nutritional vulnerability as a mediator of the association between xerostomia and frailty and reinforce the multifactorial nature of frailty. Integrating oral health and nutritional assessment into the care of older adults may facilitate the identification of individuals experiencing greater vulnerability and inform multidisciplinary strategies to promote healthy ageing. Longitudinal studies are needed to confirm the temporal direction of the observed associations and investigate other potential pathways linking xerostomia and frailty**.**

## Data Availability

The raw data supporting the conclusions of this article will be made available by the authors, without undue reservation.
