# Supplementary material for: Xerostomia symptom severity and frailty among community-dwelling older adults: a survey-weighted structural equation modeling analysis
Source: Front Dent Med. 2026 Oct 14;7:1941262. doi: 10.3389/fdmed.2026.1941262 (PMC13713752; doi:10.3389/fdmed.2026.1941262)
Supplement: Supplementary file 1 [file Supplementaryfile1.pdf]

Supplementary Table S1. Sensitivity analysis of the structural equation model additionally adjusted for municipality.

Pathway	B	95% CI	Standardized β	P-value
**Direct effects**				
SXI score → nutritional vulnerability	0.146	−0.069 to 0.361	0.179	0.169
Nutritional vulnerability → EFS score	0.232	0.057 to 0.407	0.212	**0.012**
SXI score → EFS score	0.128	0.002 to 0.254	0.144	**0.047**
**Indirect effect through nutritional vulnerability**				
SXI score → nutritional vulnerability → EFS score	0.034	−0.022 to 0.090	0.038	0.214
**Total effect on frailty**				
SXI score → EFS score	0.162	0.009 to 0.316	0.182	**0.040**

Note: Total analytical sample: n = 398. B, unstandardized coefficient; β, standardized coefficient; CI, confidence interval; EFS, Edmonton Frail Scale; SXI, Summated Xerostomia Inventory. Higher SXI scores indicate greater xerostomia symptom severity. Nutritional vulnerability was calculated as 14 minus the Mini Nutritional Assessment–Short Form score, with higher values indicating poorer nutritional status. The model was adjusted for age, sex, educational attainment, and municipality. Municipality was coded as Bauru = 0 and Boraceia = 1. Indirect effects were calculated as the products of the corresponding pathway coefficients. The model was estimated using survey weights and linearized standard errors, accounting for clustering in the sampling design. The model explained 9.7% of the variance in nutritional vulnerability and 15.9% of the variance in the EFS score.
