# Supplementary material for: Xerostomia symptom severity and frailty among community-dwelling older adults: a survey-weighted structural equation modeling analysis
Source: Front Dent Med. 2026 Oct 14;7:1941262. doi: 10.3389/fdmed.2026.1941262 (PMC13713752; doi:10.3389/fdmed.2026.1941262)
Supplement: Supplementary file 2 [file Supplementaryfile2.pdf]

Supplementary Table S2. Sensitivity analysis using the EFS score excluding its nutritional component.

Pathway	B	95% CI	Standardized β	P-value
SXI score → nutritional vulnerability	0.146	−0.073 to 0.364	0.179	0.177
Nutritional vulnerability → modified EFS score	0.129	−0.043 to 0.302	0.122	0.132
SXI score → modified EFS score: direct effect	0.123	0.014 to 0.231	0.142	**0.029**
SXI score → nutritional vulnerability → modified EFS score: indirect effect	0.019	−0.021 to 0.058	0.022	0.328
SXI score → modified EFS score: total effect	0.141	0.021 to 0.262	0.163	**0.024**

Note: Total analytical sample: n = 398. The modified EFS score excluded the nutritional domain. The model was estimated using survey weights and linearized standard errors, accounting for clustering in the sampling design. Models were adjusted for age, sex, and educational attainment.
