# Supplementary material for: Xerostomia symptom severity and frailty among community-dwelling older adults: a survey-weighted structural equation modeling analysis
Source: Front Dent Med. 2026 Oct 14;7:1941262. doi: 10.3389/fdmed.2026.1941262 (PMC13713752; doi:10.3389/fdmed.2026.1941262)
Supplement: Supplementary file 3 [file Supplementaryfile3.pdf]

Supplementary Table S3. Completeness of study variables and derivation of the analytical sample.

Stage or variable	Available, n	Missing, n
Initial study sample	437	—
Complete SXI score	409	28
Complete MNA-SF score	412	25
Complete EFS score	409	28
Complete age information	418	19
Complete sex information	419	18
Complete educational attainment	418	19
Complete information for all three scales	399	38
Final complete-case analytical sample	398	39
